# Association of arm circumference with the prevalence of gallstones in United States adults: a retrospective analysis on US National Health and Nutrition Examination Survey

**DOI:** 10.3389/fmed.2025.1511637

**Published:** 2025-01-24

**Authors:** Jianjun Wang, Xi Chen, Wei He, Xintao Zeng, Pei Yang, Jianping Gong, Decai Wang

**Affiliations:** ^1^Department of Hepatobiliary Surgery, The Second Affiliated Hospital of Chongqing Medical University, Chongqing, China; ^2^Department of Hepatobiliary Surgery, Mianyang Central Hospital, School of Medicine, University of Electronic Science and Technology of China, Mianyang, China; ^3^Department of Stomatology, Mianyang Central Hospital, School of Medicine, University of Electronic Science and Technology of China, Mianyang, China; ^4^Department of Urology, Mianyang Central Hospital, School of Medicine, University of Electronic Science and Technology of China, Mianyang, China

**Keywords:** gallstone disease, arm circumference, metabolic disorder, cross-sectional study, NHANES

## Abstract

**Background:**

Arm circumference (AC) is a measure of nutritional status and an indicator of the risk of developing diseases, such as metabolic disorders. However, its relationship with the prevalence of gallstone disease (GS), a metabolic disorder, is unknown. Consequently, this research sought to investigate the relationship between AC and the prevalence of GS among the general adult population in America.

**Methods:**

Participant data were extracted from the National Health and Nutrition Examination Survey (NHANES) 2017–2020 cycle. GS was defined based on self-reported medical history. AC was measured following standardized protocols as the primary exposure variable. Multivariable logistic regression models were employed to assess the association between AC and GS. Dose–response relationships were evaluated using generalized additive models with smoothed curve fitting, and subgroup analyses were conducted to explore effect modification by key covariates such as age, sex, race, hypertension, diabetes, and body mass index.

**Results:**

Overall, a total of 8,081 participants were included in this study, with 849 reporting a history of GS. After accounting for potential confounders, we discovered that each centimeter increase in AC was linked to an 8% rise in the prevalence of GS (Odd ratio = 1.08, 95% confidence interval: 1.07–1.10). Dose–response curves demonstrated a positive linear relationship between AC and the prevalence of GS, which, according to the results of the subgroup analyses, was consistent in the vast majority of subgroups, although there were subtle differences.

**Conclusion:**

AC exhibited a linear and positive association with the prevalence of GS. Although a causal relationship between AC and the prevalence of GS could not be established, our study provides strong new support for the potential role of AC in the health assessments of adult populations.

## 1 Introduction

Gallstone disease (GS) is a common gastrointestinal disease worldwide. Epidemiological surveys have revealed that the prevalence of GS among adults in the United States (US) and Europe is approximately 5–25% (1, 2), whereas among Asians, it is relatively low (3). The annual healthcare cost associated with GS in the US is approximately $6 billion (4), placing a significant burden on public health. Most patients with GS are asymptomatic and are diagnosed through physical examination. A small number of patients may experience abdominal pain, loss of appetite, nausea, and vomiting. In a very small number of patients, GS involving the common bile duct can lead to cholangitis and pancreatitis, which can be life-threatening in severe cases (5, 6). Previous studies have reported the potential mechanisms and risk factors for developing GS; however, validated indicators for predicting or evaluating the risk of developing GS are lacking.

The arm circumference (AC) is a simple, direct, cost-effective, and noninvasive indicator of nutritional status and risk of developing diseases. AC reflects an individual’s muscle mass and fat reserves, particularly the muscle-to-fat ratio in the upper arm area. In developing countries, AC has been utilized to evaluate the nutritional status of children and adolescents, with a small AC value indicating that a child or adolescent may be malnourished or have stunted growth (7–10). Moreover, a low AC value is linked to a higher risk of mortality in patients with cirrhosis and cardiac failure, those undergoing hemodialysis, and the elderly (11–15). Recent studies have shown that AC can be used as a novel bioindicator to assess obesity, insulin resistance (IR), metabolic syndrome (MetS), and non-alcoholic fatty liver disease (NAFLD) in adults (16–19).

Although waist circumference (WC) is an established indicator of central obesity and metabolic health, AC provides complementary information about body composition, particularly in terms of the muscle-to-fat ratio and upper extremity nutritional status. Unlike WC, which primarily reflects abdominal fat distribution, AC captures a broader physiological profile, including peripheral muscle and fat reserves. This distinction is important when exploring conditions like GS, where factors beyond central obesity may contribute to disease risk. Furthermore, there are specific clinical scenarios where accurate WC measurement may not be feasible, such as in individuals with abdominal deformities, surgical scars, or extreme obesity. In such cases, AC offers a practical and reliable alternative for assessing body composition.

However, the relationship between AC and GS remains unclear. Given that GS is, to some extent, a metabolic disorder, we hypothesized a link between AC and GS. This study aimed to evaluate the relationship between AC and the prevalence of GS in US adults, utilizing data from the National Health and Nutrition Examination Survey (NHANES) to provide evidence of AC’s potential role in adult health assessments. By focusing on AC as a less explored but complementary bioindicator, we sought to uncover novel insights into the relationship between body composition and GS, thereby contributing to a more comprehensive understanding of metabolic health.

## 2 Materials and methods

### 2.1 Study design and participants

Participant information was extracted from NHANES, an open-source database officially maintained by the Centers for Disease Control and Prevention. The NHANES provides valuable data support for disease prevention, public health policy development, clinical practice, and individualized medicine and plays a very important role in nutritional assessment, research, and education. NHANES is updated biennially with a population of approximately 10,000 at a time. NHANES employs a stratified multistage probability sampling design to ensure the representativeness of its sample at the national level. This approach involves several key steps: first, the population is stratified into distinct groups based on relevant characteristics; then, primary sampling units (PSUs) are selected, followed by the identification and selection of secondary sampling units (SSUs) within each PSU. In the next phase, households are randomly sampled from the SSUs. Additionally, NHANES incorporates an oversampling strategy to ensure that specific subgroups, such as minority populations or those with specific health conditions, are adequately represented in the final sample. This comprehensive sampling methodology ensures that the data collected are reflective of the diverse U.S. population, providing a robust basis for national health assessments. Due to the coronavirus disease 2019, NHANES was suspended in March 2020. Data from the 2017–2020 cycle were utilized for the analysis as the GS questionnaire was exclusively available during these years. The questionnaire was conducted exclusively among adults who were 20 years of age or older.

### 2.2 Definition of data

AC was an exposure variable in this study. For AC measurement, the participant stood upright with the arm hanging loosely, and the examiner stood facing the participant’s right side, comfortably rolling up the tape measure at the midpoint of the upper arm without pressing on the skin. The tape was placed perpendicular to the long axis of the upper arm with the ends overlapping, and the resultant tape was located on the side of the arm. The AC was recorded to the nearest millimeter (20).

### 2.3 Data collection

A questionnaire was used to assess patients with GS. GS in the NHANES dataset (2017–2020) was defined based on self-reported medical history, where participants were asked if they had ever been told by a doctor that they had gallstones. The main covariates included in this study were demographic variables, comorbidities, dietary intake, laboratory findings, and other parameters. Detailed results are presented in Table 1. All study participants were asked to complete two 24-h dietary recalls, which were conducted on separate, non-consecutive days within the same survey cycle. To provide a more accurate estimate of dietary intake, the average of the two recall days was calculated and used in the subsequent analysis. This method helps account for day-to-day variability in dietary consumption, offering a more reliable representation of participants’ typical food intake. Detailed procedures for measuring all covariates are available on the official NHANES website. The official link is: www.cdc.gov/nchs/nhanes. All NHANES protocols adhered to the U.S. Department of Health and Human Services Policy for the Protection of Human Research Subjects and underwent annual review and standardization by the NCHS Research Ethics Review Board. All NHANES participants signed an informed consent form. Therefore, no additional permission or ethical review was required for this study.

**Table 1:** Covariates extracted from NHANES (2017–2020).

Items	Composition
Demographic variables	Age, Gender, Race
Comorbidities	Hypertension, Diabetes, Asthma, CHD, Cancer
Laboratory results	TG, TC, HDL-C, LDL-C, ALT, AST, HbA1c, Ferritin
Dietary intake factors	Total sugar, Total Kcal, Total fat, Total water
Others	AC, BMI, Physical activity, Marital status, Alcohol consumption, Education level, Smoking status, PIR

NHANES, National Health and Nutrition Examination Survey; CHD, coronary heart disease; TG, triglyceride; TC, total cholesterol; HDL-C, high-density lipoprotein cholesterol; LDL-C, low-density lipoprotein cholesterol; ALT, alanine aminotransferase; AST, aspartate aminotransferase; HbA1c, hemoglobin A1c; AC, arm circumference; BMI, body mass index; PIR, poverty income ratio.

### 2.4 Outcome indicators

The outcome variable was the prevalence of GS.

### 2.5 Statistical analysis

Categorical variables were expressed as numbers and percentages (%), and comparisons between groups were made using Pearson’s *χ*2 test or Fisher’s exact test. Continuous variables are expressed as medians and interquartile ranges, and comparisons of continuous variables between groups were performed using a *t*-test or one-way ANOVA. Following these guidelines, multivariate logistic regression models were performed to examine the association between AC and the prevalence of GS across various model specifications: Model 1, which did not include adjustments for covariates; Model 2, which accounted for age, sex, and race, and Model 3, which was additionally adjusted for physical activity, smoking status, diabetes, hypertension, coronary heart disease (CHD), triglyceride, alanine aminotransferase, total kcal intake. The relationship between AC and the prevalence of GS was further assessed using a generalized additive model regression combined with smoothed curve fitting (penalized spline method). When a nonlinear relationship was observed, a logarithmic likelihood ratio test was employed to determine the inflection point values. Subgroup analyses were performed according to age, sex, race, hypertension, diabetes, and body mass index (BMI). A *p*-value of less than 0.05 was considered statistically significant. All analyses were conducted using R version 4.0.2^1^ (R Foundation) and Empower software^2^ (X&Y Solutions, Inc., Boston, MA, USA).

## 3 Results

### 3.1 Participants’ baseline characteristics

**Figure 1:**
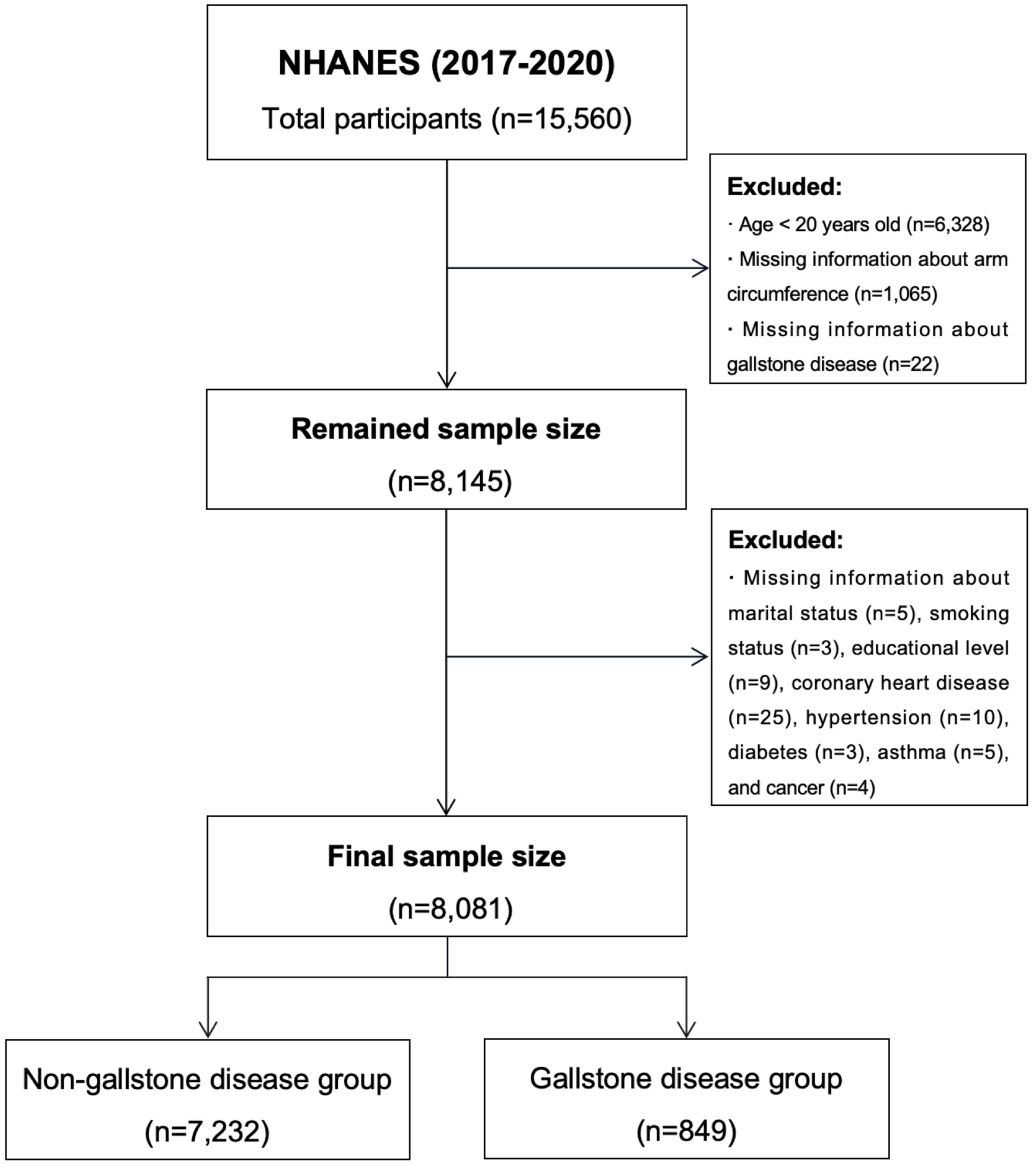
Flowchart for participants from NHANES (2017–2020).

The study included a total of 8,081 participants, of whom 849 had a history of GS. Figure 1 illustrates the participant selection process. Table 2 presents the baseline characteristics of the study participants. The GS group exhibited higher AC values compared to the non-GS group [33.55 (5.20) vs. 35.27 (6.00), *p* < 0.001]. The patients in the GS group were older; had a higher BMI; and a higher prevalence of hypertension, diabetes, asthma, CHD, and cancer. Moreover, significant differences were observed between the two groups in terms of sex, race, physical activity, marital status, alcohol consumption, smoking status, total calory intake, total fat intake, and total triglycerides, hemoglobin A1c, and Ferritin levels (All *p* < 0.05).

**Table 2:** Baseline characteristics of participants (weighted).

Characteristic	All (*n* = 8,081)	Non-GS group (*n* = 7,232)	GS group (*n* = 849)	*p*-value
*N*	213711302.55	190551072.20	23160230.34	
Age (years), mean (SD)	48.17 (17.08)	47.14 (16.97)	56.71 (15.46)	<0.001
Female (%)	51.9	49.3	68.9	<0.001
AC (cm), mean (SD)	33.73 (5.32)	33.55 (5.20)	35.27 (6.00)	<0.001
BMI, mean (SD)	29.81 (7.26)	29.41 (6.95)	33.15 (8.77)	<0.001
Race (%)				
Non-Hispanic White	62.9	62.2	68.9	0.02
Non-Hispanic Black	11.4	11.9	7.3	
Mexican American	15.9	16.0	15.1	
Other race	9.8	9.9	8.7	
Physical activity (%)				
Never	23.7	22.9	30.4	<0.001
Moderate	34.3	33.7	39.4	
Vigorous	42.0	43.3	30.2	
Marital status (%)				
Cohabitation	62.8	62.6	64.5	<0.001
Solitude	18.1	17.5	23.2	
Never married	19.1	19.9	12.3	
Alcohol consumption (%)				
Never	6.4	6.3	7.1	<0.001
Ever	15.3	13.9	26.9	
Now	74.7	76.2	62.1	
Unclear	3.6	3.6	4.0	
Education level (%)				
Less than high school	10.4	10.5	9.2	0.14
High school	27.0	26.5	31.1	
More than high school	62.6	63.0	59.7	
Hypertension (%)	32.0	30.0	48.7	<0.001
Diabetes (%)	11.3	10.3	20.0	<0.001
Asthma (%)	15.4	15.1	18.2	0.13
CHD (%)	4.1	3.7	7.1	0.001
Cancers (%)	11.1	10.2	18.6	<0.001
Smoking status (%)				
Never	57.5	58.1	52.2	0.01
Ever	25.6	24.9	32.2	
Now	16.9	17.0	15.6	
PIR				
< 1.3	16.5	16.5	16.1	0.006
≥ 1.3–<3.5	31.1	30.1	39.1	
≥ 3.5	41.5	42.2	35.8	
Unclear	10.9	11.1	9.0	
Total Sugar, mean (SD)	106.52 (78.42)	106.74 (78.65)	104.71 (76.58)	0.58
Total Kcal, mean (SD)	2176.10 (991.73)	2204.17 (1003.40)	1944.24 (855.50)	<0.001
Total Fat, mean (SD)	89.74 (48.81)	90.80 (49.31)	80.97 (43.51)	0.001
Total Water, mean (SD)	2497.22 (2384.69)	2513.38 (2372.27)	2363.89 (2482.38)	0.20
TG (mmol/L), mean (SD)	1.58 (1.15)	1.57 (1.17)	1.67 (1.01)	0.04
TC (mmol/L), mean (SD)	4.86 (1.06)	4.86 (1.06)	4.86 (1.05)	0.96
HDL-C (mmol/L), mean (SD)	1.39 (0.41)	1.39 (0.41)	1.37 (0.39)	0.31
LDL-C (mmol/L), mean (SD)	2.89 (0.93)	2.89 (0.92)	2.88 (0.98)	0.95
ALT (U/L), mean (SD)	22.69 (16.94)	22.76 (17.07)	22.07 (15.83)	0.47
AST (U/L), mean (SD)	21.79 (12.70)	21.87 (12.79)	21.13 (11.95)	0.20
HbA1c (%), mean (SD)	5.68 (0.95)	5.65 (0.93)	5.92 (1.08)	<0.001
Ferritin (ng/ml), mean (SD)	147.69 (161.34)	149.64 (163.05)	131.81 (145.81)	0.01

“*N*” represents the weighted total number of participants.

AC, arm circumference; BMI, body mass index; CHD, coronary heart disease; PIR, poverty income ratio; TG, triglyceride; TC, total cholesterol; HDL-C, high-density lipoprotein cholesterol; LDL-C, low-density lipoprotein cholesterol; ALT, alanine aminotransferase; AST, aspartate aminotransferase; HbA1c, hemoglobin A1c.

### 3.2 Logistic regression analysis between AC with gallstone disease prevalence

The multivariable logistic regression analysis results revealed a positive correlation between high AC values and a high prevalence of GS. This relationship persisted even in the fully adjusted model (Model 3) (OR = 1.08, 95% CI: 1.07–1.10), meaning that for every 1 centimeter increase in AC, the prevalence of GS increases by 8%. When AC was converted from a continuous variable to a categorical variable (tertiles) for further analyses, we found a 2.43-fold increase in the prevalence of GS in the third tertile (OR = 2.43, 95% CI: 1.91, 3.10) compared with that in the lowest AC tertile (tertile 1). The comprehensive results are displayed in Table 3.

**Table 3:** Logistic regression analysis between AC with gallstone disease prevalence (weighted).

Characteristic	Model 1 OR (95% CI)	Model 2 OR (95% CI)	Model 3 OR (95% CI)
AC	1.06 (1.04–1.07)	1.10 (1.08–1.11)	1.08 (1.07–1.10)
Categories			
Tertile 1	1	1	1
Tertile 2	1.28 (0.95–1.72)	1.64 (1.20–2.23)	1.47 (1.07–2.00)
Tertile 3	1.83 (1.48–2.26)	3.05 (2.44–3.81)	2.43 (1.91–3.10)

Model 1 = no covariates were adjusted.

Model 2 = Model 1 + age, gender, and race were adjusted.

Model 3 = Model 2 + physical activity, smoking status, diabetes, hypertension, coronary heart disease, triglyceride, alanine aminotransferase, total kcal intake were adjusted.

### 3.3 Dose–response and threshold effects of AC on GS prevalence

**Figure 2:**
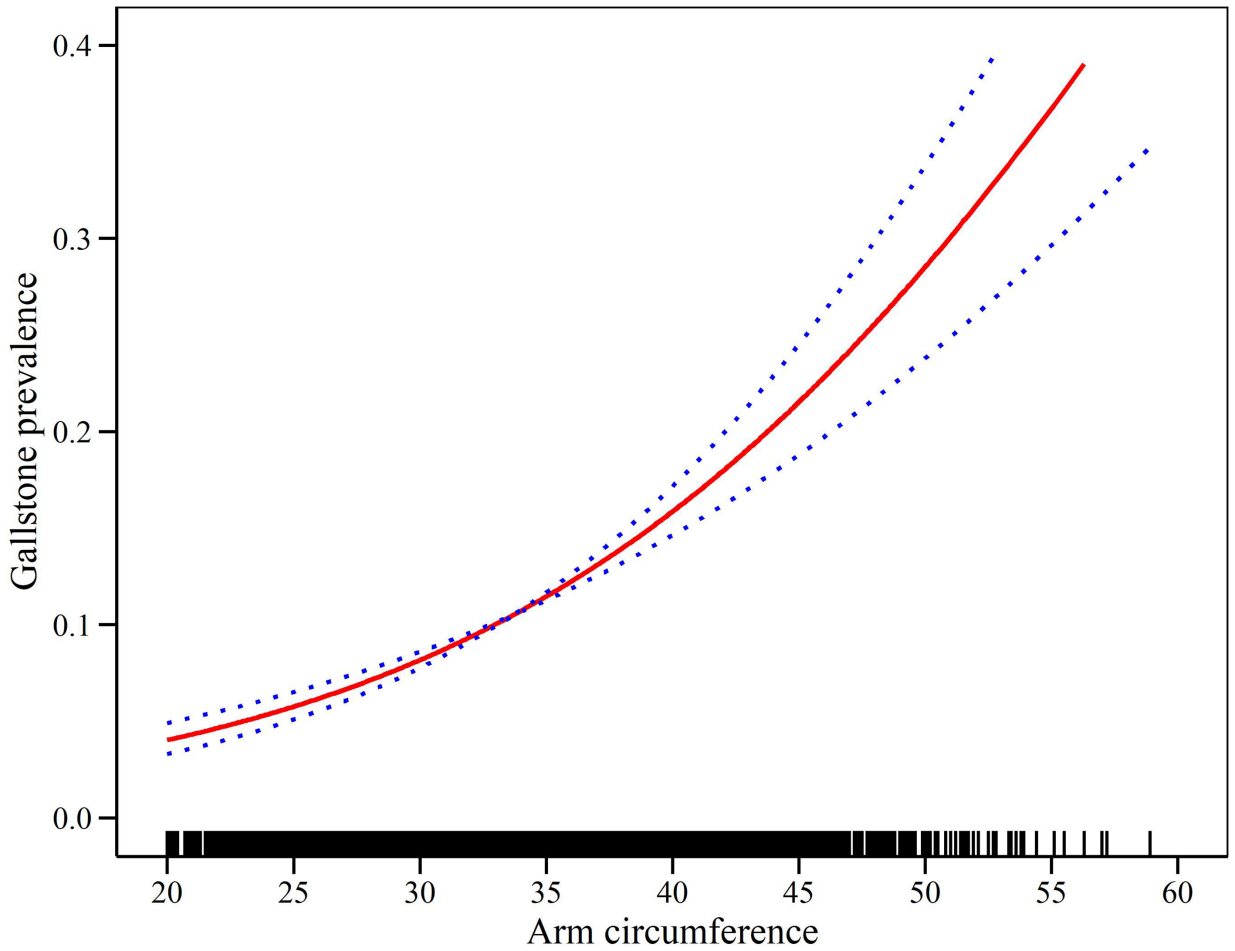
Density dose–response relationship between AC and gallstone disease prevalence. The area between the upper and lower dashed lines representes the 95% CI.

The relationship between AC and the prevalence GS was further explored using generalized additive models and smooth curve fitting. The results demonstrated a significant positive linear correlation between AC and GS prevalence (Figure 2 and Table 4). In Figure 2, the confidence intervals (CIs) around the dose–response curve narrow as AC increases from approximately 25 cm to 50 cm, reflecting greater precision and reliability in this range. For higher AC values (>50 cm), the CIs widen slightly due to fewer participants with very large AC, introducing greater variability. Nonetheless, the overall positive linear relationship between AC and gallstone disease prevalence remains robust, as supported by the logistic regression results in Table 3.

**Table 4:** Two-piecewise linear regression and logarithmic likelihood ratio test explained the threshold effect analysis of AC with gallstone disease prevalence.

AC	ULR test	PLR test	LRT test
	OR (95% CI)	OR (95% CI)	*p*-value
< 39	1.08 (1.06–1.10)	1.09 (1.06–1.11)	0.30
> = 39		1.06 (1.02–1.10)	

ULR, univariate linear regression; PLR, piecewise linear regression; LRT, logarithmic likelihood ratio test, statistically significant: p < 0.05.

### 3.4 Subgroup analysis

Subgroup analyses revealed a consistent positive association between arm circumference (AC) and the prevalence of gallstone disease (GS) across various demographic and clinical subgroups (Table 5). Stratified by age, the strongest association was observed in participants aged 20–39 years (OR: 1.11, 95% CI: 1.08–1.14), with the association attenuating in older age groups, particularly among those aged 60–80 years (OR: 1.05, 95% CI: 1.03–1.08; *P* for interaction = 0.006). When stratified by gender, the association was more pronounced in females (OR: 1.10, 95% CI: 1.08–1.11) compared to males (OR: 1.06, 95% CI: 1.02–1.09; *P* for interaction = 0.001).

**Table 5:** Subgroup analysis between AC with gallstone disease prevalence.

Characteristic	Model 1 OR (95% CI)	Model 2 OR (95% CI)	Model 3 OR (95% CI)	*P* for interaction
Stratified by age (years)				
20–39	1.10 (1.07–1.13)	1.12 (1.09–1.15)	1.11 (1.08–1.14)	0.006
40–59	1.08 (1.06–1.11)	1.11 (1.08–1.14)	1.10 (1.08–1.13)	
60–80	1.04 (1.02–1.06)	1.07 (1.04–1.09)	1.05 (1.03–1.08)	
Stratified by gender				
Female	1.08 (1.07–1.10)	1.10 (1.09–1.12)	1.10 (1.08–1.11)	0.001
Male	1.02 (0.99–1.05)	1.07 (1.04–1.10)	1.06 (1.02–1.09)	
Stratified by race				
Non-Hispanic White	1.06 (1.04–1.08)	1.10 (1.07–1.12)	1.08 (1.06–1.11)	0.36
Non-Hispanic Black	1.07 (1.04–1.09)	1.07 (1.05–1.10)	1.06 (1.04–1.10)	
Mexican American	1.05 (1.02–1.08)	1.09 (1.06–1.13)	1.08 (1.04–1.12)	
Other race	1.08 (1.04–1.12)	1.13 (1.09–1.18)	1.11 (1.06–1.16)	
Stratified by BMI				
≤ 24.9	0.92 (0.86–0.99)	0.98 (0.90–1.06)	0.99 (0.91–1.08)	0.40
25–29.9	0.91 (0.86–0.96)	1.04 (0.98–1.11)	1.03 (0.96–1.10)	
≥ 30	1.02 (1.00–1.04)	1.07 (1.05–1.09)	1.06 (1.03–1.08)	
Stratified by hypertension				
No	1.05 (1.03–1.07)	1.09 (1.07–1.11)	1.08 (1.06–1.11)	0.95
Yes	1.05 (1.03–1.07)	1.09 (1.06–1.11)	1.07 (1.05–1.09)	
Stratified by diabetes				
No	1.06 (1.04–1.07)	1.10 (1.08–1.11)	1.09 (1.07–1.11)	0.16
Yes	1.04 (1.01–1.07)	1.07 (1.04–1.10)	1.05 (1.01–1.08)	

Model 1 = no covariates were adjusted.

Model 2 = Model 1 + age, gender, and race were adjusted.

Model 3 = Model 2 + physical activity, smoking status, diabetes, hypertension, coronary heart disease, triglyceride, alanine aminotransferase, total kcal intake were adjusted.

The association remained statistically significant across all racial groups, with the highest odds observed in the “Other Race” category (OR: 1.11, 95% CI: 1.06–1.16), followed by Non-Hispanic Whites (OR: 1.08, 95% CI: 1.06–1.11; *P* for interaction = 0.36). When stratified by BMI, the relationship was strongest in participants with a BMI ≥30 (OR: 1.06, 95% CI: 1.03–1.08), while it was not statistically significant in those with a BMI ≤24.9 (OR: 0.99, 95% CI: 0.91–1.08; *P* for interaction = 0.40). Furthermore, the association persisted regardless of hypertension status, with comparable odds ratios in hypertensive (OR: 1.07, 95% CI: 1.05–1.09) and non-hypertensive participants (OR: 1.08, 95% CI: 1.06–1.11; *P* for interaction = 0.95). Stratification by diabetes status revealed a stronger association in participants without diabetes (OR: 1.09, 95% CI: 1.07–1.11) compared to those with diabetes (OR: 1.05, 95% CI: 1.01–1.08; *P* for interaction = 0.16).

In summary, the positive association between AC and GS prevalence was consistent across most subgroups, with stronger associations observed in younger participants, females, and those with higher BMI.

## 4 Discussion

This study is the first to comprehensively examine the relationship between AC and GS prevalence. We found a linear positive correlation between AC and GS prevalence, with an 8% increase in the prevalence of GS for every 1 cm increase in AC. After categorizing AC into tertiles, we observed a 2.43-fold increase in GS prevalence among individuals in the highest AC tertile than in those in the lowest AC tertile. The results of the subgroup analyses further indicated that AC showed a positive association with GS prevalence across nearly all populations, with only slight variations. Our results confirm the importance of AC as a predictor of the risk of developing GS.

While our findings are novel, it is essential to position AC within the broader context of established indicators of obesity, such as BMI and WC, which are well-recognized predictors of MetS and GS. BMI is the most commonly used measure of overall adiposity, while WC serves as a strong indicator of visceral fat accumulation, which is closely linked to metabolic abnormalities. In contrast, AC is a simple, practical, and noninvasive measure that simultaneously reflects body composition (including muscle and fat mass) and nutritional status. Importantly, our study demonstrated that the association between AC and GS prevalence remained significant even after adjusting for BMI, WC, and other metabolic confounders. This finding suggests that AC provides complementary information beyond BMI and WC, particularly in populations where assessing WC or BMI is not feasible or practical.

GS is prevalent globally, especially in developed countries, with 1.5 million people seeking medical care for GS in the US in 2015 alone (21). The pathogenesis of GS is complex and involves several factors. Earlier studies have indicated a higher prevalence of GS in females compared to males (22, 23), which may be because of estrogen-induced increase in the synthesis and secretion of hepatic cholesterol by increasing the expression of estrogen receptor 1 and G protein-coupled receptor 30 as well as decreased bile salt synthesis, and ultimately promoting GS development (24). Moreover, estrogen weakens the contractility of the gallbladder and prolongs the retention time of bile, thereby increasing the deposition of cholesterol crystals (24). Similarly, we found a higher prevalence of GS in females than in males. Advanced age is considered an important factor in the increased prevalence of GS (25, 26). Similarly, our study found that the mean age of individuals without GS was 50 years (ranging from 35 to 63), while for those with GS, it was 60 years (ranging from 46 to 71). With increasing age, the composition of bile changes, and the amount of cholesterol in bile may increase, whereas the amount of bile acid salts may decrease, leading to an increased risk of cholesterol crystal formation and, consequently, an increased incidence of GS (27).

Moreover, the motor function of the gallbladder may weaken, leading to a longer bile retention time, which increases the risk of developing GS (28, 29). However, recent studies have highlighted that metabolic factors, such as obesity and diabetes, significantly influence the prevalence of GS, particularly among younger populations (30, 31). Genetic factors are also important risk factors for GS, with its prevalence varying according to race. In developed countries, the prevalence of GS is significantly higher in white populations than in black populations (28). For example, an epidemiological survey found that, in the US, the prevalence of GS was 16.6% among white women compared with 13.9% among black women and 8.6% among white men, compared with 5.3% among black men (32). This may be related to the differences in dietary habits, lifestyles, and healthcare among the different races. For example, white populations have greater access to high-calorie food, which may have contributed to a higher prevalence of GS. Similarly, our study found that the prevalence of GS is highest among non-Hispanic white people compared with other populations.

Recently, a growing body of research has identified a strong correlation between the rising incidence of GS and the high prevalence of MetS (26, 33, 34). Among them, IR is a key mechanism underlying the increased incidence of GS. In hepatocytes, IR promotes cholesterol secretion by causing abnormal expression of the transcription factor forkhead box protein O1 through the action of ABCG5 and ABCG8 (35), which may explain the higher prevalence of GS among individuals with diabetes. The prevalence of GS was also higher among individuals with obesity than among those without obesity (26). The main reason for this is obesity-induced metabolic changes, such as increased bile secretion from the liver, hyperlipidemia, reduced intestinal motility, and dysfunctions in various metabolic organs, including the liver and gallbladder (36). Further, our study revealed that the mean BMI of participants in the GS group was higher than that of those in the non-GS group [31.8 (27.7–37.7) vs. 28.5 (24.6–33.3), *p* < 0.001]. Notably, the prevalence of GS is increasing among the younger population due to the increased intake of high-calorie foods as society develops, and economic standards increase, as well as increased physical inactivity (37).

AC is a simple and noninvasive measure of great value for evaluating the nutritional status of the body and the risk of disease development. The connection between AC, MetS, and related diseases has been thoroughly investigated (23, 26, 33, 38–40). In a cross-sectional study including 2,397 patients, Wang et al. discovered a positive association between AC and both hepatic steatosis and fibrosis and that this positive association was consistent across sex and race (Mexican Americans, other Hispanics, non-Hispanic white people, Black population, Asians, and other races) (41). Xiao et al. investigated the relationship between AC and both all-cause and cardiovascular-related mortality in patients with diabetes (20). They identified a significant negative correlation between AC and both all-cause and cardiovascular-related mortality in this population (20). In a study by Chao et al. that included 11,527 participants, AC showed a significant positive association with HOMA-IR, an indicator of IR (42).

As mentioned previously, the pathogenesis of GS is closely related to metabolic abnormalities. However, the relationship between the AC and GS has not yet been effectively explored. In this study, we examined the association between AC and GS prevalence. We discovered that AC showed a linear positive correlation with the prevalence of GS and that this relationship was maintained across different subgroups, although there were subtle differences. Our findings offer new insights into the potential utility of AC in evaluating adult health.

Our study has several strengths. First, this study is the first to clarify the relationship between AC and GS prevalence. Second, NHANES provided a representative sample of US participants who followed a meticulously designed study protocol and underwent stringent quality control and assurance, thereby ensuring the reliability of our conclusions. Third, the comprehensive demographic and metabolic data, along with detailed follow-up information, enabled us to account for potential confounders. Finally, we also performed subgroup analyses to ensure the generalizability of our results to a wider population.

Nevertheless, our study has certain limitations. First, as this was a cross-sectional study, we were unable to establish a causal relationship between AC and GS. While causal inference methods, such as propensity score matching or instrumental variable analysis, could strengthen causal interpretations, the nature of our study and data limitations restricted their application. Nonetheless, sensitivity analyses demonstrated consistent results, underscoring the robustness of our findings. Future longitudinal or Mendelian randomization studies are warranted to explore causal relationships further. Second, GS was diagnosed based on a questionnaire survey, which might have been influenced by recall bias. Third, owing to the design of the NHANES, we could only measure AC at a single time point at baseline; however, AC may change over time. Finally, although we adjusted for many possible confounders, we could not eliminate the potential impact of unmeasured confounders.

Despite these limitations, our study provides new insights into the relationship between AC and GS prevalence. Compared with BMI and WC, AC represents a practical and complementary indicator that may help assess GS risk, particularly in resource-limited or large-scale screening settings. In the future, we plan to conduct prospective cohort studies to evaluate the longitudinal relationship between AC and GS incidence, as well as comparative analyses to explore the relative predictive value of AC, WC, and BMI in identifying individuals at high risk of GS. These efforts will further clarify the utility of AC in metabolic and gallstone disease management.

## 5 Conclusion

Our study is the first to uncover the link between AC and GS prevalence. Specifically, there was a linear positive correlation between AC and GS prevalence, with GS prevalence increasing by 8% for each 1 cm rise in AC. While we could not establish a causal relationship between AC and GS prevalence, the result remains noteworthy.

## Data Availability

The original contributions presented in the study are included in the article/supplementary material, further inquiries can be directed to the corresponding authors.

## Footnotes

^1^ http://www.R-project.org

^2^ www.empowerstats.com
